# Corrosion-Induced Crack Quantification in Reinforced Concrete with Portland and Slag-Blended Cement Under Accelerated Exposure

**DOI:** 10.3390/ma19112278

**Published:** 2026-05-28

**Authors:** Bar Krauze, Yuri Ribakov, Gili Lifshitz Sherzer

**Affiliations:** Department of Civil Engineering, Ariel University, Ariel 40700, Israel; barkr@ariel.ac.il (B.K.); gilil@ariel.ac.il (G.L.S.)

**Keywords:** corrosion, direct current, reinforced concrete, carbon footprint, concrete degradation, blast furnace slag-blended cement

## Abstract

Optimizing the service life of reinforced concrete structures requires replacing traditional Portland Cement (PC) with Slag-Blended Cements (SBCs) that offer refined pore networks, which are vital for inhibiting the propagation of corrosion-induced cracks. In this study, we propose an integrated framework combining Direct Current (DC)-accelerated corrosion tests with computational quantification of cracking. For comparison purposes, the concrete samples with similar compressive strengths (~60 MPa), obtained after 65 days from the mixing process, were exposed to impressed currents while the evolution of cracks was monitored using image processing in MATLAB. It was found that the slag-blended cement significantly delayed the appearance of the crack, which occurred at 141 h, compared with 57 to 70 h for the PC specimen. The delay in corrosion damage initiation by SBC is 1.7 times higher than that by PC. In terms of damage severity, SBC reduced both the total crack lengths by 56% (83 mm for SBC and 189 mm for PC) and the maximum crack width by 22% (0.70 mm for SBC and 0.90 mm for PC). After 111 h of corrosion under the same conditions, the SBC still retained its ability to reduce the crack length (188 mm), whereas PC formed 270 mm cracks. These findings provide a basis for future calibration of sophisticated mesoscale fracture models, such as the Lattice Discrete Particle Method (LDPM) and the Finite Discrete Element Method (FDEM), as well as for creating data sets for future data-driven durability assessment.

## 1. Introduction

The engineering community faces two closely linked challenges in Reinforced Concrete (RC) infrastructure: reducing the environmental footprint of PC production while also addressing the widespread durability problems associated with steel reinforcement corrosion. This has led to increasing interest in alternative binders that can provide both enhanced durability and a lower environmental impact [1]. The rate of corrosion can be greatly affected by the environment to which the structure is subjected. In reinforced concrete structures, electrochemical corrosion is the dominant factor that determines how long a structure can withstand environmental conditions. If the passive film on the steel reinforcement is disrupted by chloride ions or carbon dioxide intrusion, the expansive corrosion by-products will deposit at the steel–concrete interface [2]. Since corrosion products occupy a large volume (2 to 6 times the steel’s initial volume), they will act as a radial tensile pressure on the surrounding concrete due to the constraints imposed by the concrete [3,4,5,6]. Since concrete is a quasi-brittle material, these stresses tend to result in the formation and propagation of cracks within the concrete cover [7,8]. This cracking is not only a consequence of damage but also accelerates the transport of aggressive agents and thus reinforces a self-perpetuating cycle of deterioration [9].

### 1.1. Mechanisms of Corrosion in Cementitious Materials

The corrosion behavior of PC and SBC concretes is very different due to differences in pore structure and electrochemical reactions. In terms of binders, Ground Granulated Blast-furnace Slag (GGBS) is commonly used as a secondary cementing agent. Compared with PC, the hydration of slag consumes calcium hydroxide and promotes formation of secondary Calcium Silicate Hydrate (C–S–H) with a lower Ca/Si ratio [10]. In line with this finding, studies on blast-furnace slag-mixed concrete exposed to wetting and drying cycles in seawater have also confirmed improvements in chloride binding capacity, reduced chloride penetration, and refined pores, thus supporting the beneficial effect of slag on transport properties [11]. The refinement of the microstructure leads to a finer distribution of pore sizes and reduced permeability [12]. Apart from the effect of transport resistance, the corrosion caused by chlorides is further influenced by the number of free chlorides present in the solution. In addition, not all the ingressing chlorides are present in the solution. In the case of concretes with a significant amount of Tri-Calcium Aluminate (C3A) present in the cement, the binding of chloride is achieved by the formation of Friedel’s salt in the Aluminate Ferrite Mono-substituent (AFm) phases [13]. These microstructural characteristics are also reflected in the steel–concrete Interfacial Transition Zone (ITZ). While the interface in PC is often characterized as a porous, Calcium Hydroxide (CH)-rich region, slag-containing mixtures tend to develop a denser interface over time. Research by Duraman and Richardson [14] found that mixes containing GGBS had low CH content and decreasing porosity with time, while the steeper profiles for unreacted slag at the interface indicated a denser ITZ compared to those found in PC. In the current research, ITZ thickness has been estimated based on their work and an analytical formulation was used to estimate the time to corrosion-induced cover cracking. Its consistency was then evaluated with experimental data. These factors provide the rationale for the current research, which examines the effect of slag on cracking evolution.

### 1.2. Experimental Methods for Accelerated Corrosion

As natural corrosion is a slow process that occurs over a long period of time, an accelerated corrosion test is often used in laboratory studies. The most common test is the impressed-current test, in which an anodic current is applied to the reinforcement [15,16]. The theoretical basis for the impressed current test is Faraday’s law, which assumes a linear relationship between the charge passed and mass loss [16]. Nevertheless, although Faraday’s law suggests a theoretical loss of mass, the fit of the results obtained in experiments may differ. Peng et al. [17] used Digital Image Correlation (DIC) to analyze these discrepancies, which arise from the non-uniformity of the corrosion layer, leading to strain concentration along the reinforcement bar. In practice, this non-uniformity implies that the test setup must be carefully calibrated to connect corrosion kinetics to specific mechanical outcomes, such as bond–slip relationships. These mechanical effects are important for the understanding of the performance of reinforced concrete with unconventional binder materials, including ground granulated blast furnace slag, with ITZ microstructure and density significantly determining corrosion rate [14]. In addition, bond deterioration is a critical part of the mechanical effects of steel corrosion. As such, testing bond-related behavior, including failure modes and load–slip curves, has also been conducted on slag-based concrete [18]. Even so, studies directly comparing the effects of binder type on the onset and development of corrosion-induced cracks are relatively scarce. The present study addresses this deficiency by comparing PC and SBC directly subjected to DC tests while monitoring crack formation and development using imaging techniques.

### 1.3. Approaches for Monitoring Damage Progression; In Cement-Based Materials

The accuracy of corrosion evaluation can be improved by using real-time monitoring methods to track the progression of corrosion damage. In RC structures, corrosion-induced damage has been observed mainly as mechanical effects, owing to the volumetric expansion of corrosion products at the interface transition zone. To accurately measure the corrosion damage state, Andrade et al. used a strain sensor to establish a critical durability parameter for the initiation process [15]. However, strain gauges provide only localized deformation measurements and do not quantify crack geometry or spatial distribution. In conjunction with surface observations, Non-Destructive Evaluation (NDE) methods also play an important role in detecting internal defects. In recent years, linear and nonlinear ultrasonic techniques have shown high sensitivity in detecting distributed micro- and macroscale damage in concrete [19]. These ultrasonic techniques can be seen to complement existing techniques such as Acoustic Emission (AE), which provides time-dependent information on fracture and damage evolution [20], and the Impact-Echo (IE) method for detecting flaws and voids in materials [21]. While these techniques can detect physical defects, laser-induced breakdown spectroscopy has been used to investigate chloride-induced corrosion by analyzing the elemental content of the concrete sample [22]. To assess the mechanical impact of this chemical invasion on concrete, modern techniques such as X-ray computed microtomography (microCT) and coupled multi-electrode arrays are used to characterize damage morphology. Research conducted using these methods has proven that the choice of materials used affects the pattern of corrosion significantly. In particular, slag mortars tend to exhibit localized pitting, as opposed to homogeneous corrosion experienced by regular binders [23]. Once localized defects are detected, fast detection techniques, such as Lamb-wave-based 3D velocity contours, can be used to identify defects, such as delaminations and surface cracks [24]. For monitoring damage over a larger surface area, Li et al. [25] demonstrated that DIC can be used to map surface strain fields during corrosion and to monitor deformation over an entire observation area. However, DIC typically requires a dedicated optical setup and specialized image processing for each measurement configuration. López-Pérez et al. [26] used image processing methods, including segmentation and skeletonization, to identify microcracks in SEM images and assess their spatial orientations. Still, their quantification procedure remains manually controlled. The damage evolution was analyzed using an automated image processing process based on MATLAB software that would allow for the extraction of the fracture patterns observed in sequential images taken during the test. This technique allows higher time resolution, which is normally limited by high-performance DIC systems.

### 1.4. Training AI Models Using Experimentally Validated Numerical Simulations

One of the major limiting factors in durability evaluation is a lack of associated data linking material composition to outcomes. To bridge the gap between limited experimental data and the high data requirements of artificial intelligence, a hybrid approach combining numerical simulation is often used. For instance, recent studies illustrate how physics-based numerical models can be used to generate controlled damage scenarios for deep-learning-based damage localization and severity estimation [27]. To generate high-quality synthetic data, multiscale and mesoscale fracture modeling frameworks are important [28]. In concrete fracture modeling, particle- and lattice-based approaches are well established, including random-particle models [29] and 3D lattice fracture models [30], which represent cracking as an emergent process. One such approach is the Lattice Discrete Particle Model (LDPM), which has been used to simulate tensile fracture and multiaxial loading in concrete materials [31]. Building on this, upscaling approaches have been proposed to estimate LDPM parameters from micro-level chemical and mechanical models [32]. Another method for simulating fracture initiation and propagation is the Finite-Discrete Element Method (FDEM) [33]. When combined with microscale characterization, as in [34], it can provide a more detailed representation of heterogeneity-driven cracking. Recent research has also compared LDPM and FDEM approaches in terms of their ability to capture size effects in structural response [35]. Once validated, such numerical models can be used to generate synthetic datasets for subsequent data-driven analysis. Nonetheless, the validity of such AI-based systems and their ability to overcome the limitations that commonly arise from the issues of segmentation and generalization [36] are intrinsically contingent upon the accuracy of the numerical model used. Through the provision of highly accurate quantitative data regarding crack growth due to corrosion, the suggested experiment can offer crucial support for such efforts. In parallel, physics-informed neural networks provide a framework for combining data and governing equations in forward and inverse problems, including model inversion and parameter identification [37]. An example of such a model is a Physics-Informed Neural Network (PINN), which is designed to predict the flexural capacity of corroded RC beams. The physics of the problem are integrated into the loss function in this model. This approach leads to better predictions and generalization than conventional approaches [38]. Future work will use the experimental results from this paper to calibrate and validate numerical models, which, in turn, will be used to generate synthetic data for calibrating Artificial Intelligence (AI)-based predictive models.

## 2. Research Aims, Scope and Novelty

While there are well-established methodologies to facilitate electrochemical enhancement and mechanical behavior evaluation, one of the challenges currently faced in this body of work is the lack of correlated high-resolution datasets that can be used to bridge experimentation and predictive computational models [39]. The separation between the electrochemical and mechanical effect of corrosion hinders efforts to link the electrochemical “cause” (corrosion current) to the mechanical “effect” (crack formation and growth), according to the current literature on the topic [6,39]. In addition, despite the considerable advances in computational methods such as LDPM and FDEM, their use is limited by the lack of experimental data with sufficient resolution for validation [40,41].

The present paper addresses that gap by providing an experimental–computational pipeline for highly detailed monitoring of RC durability. The unique feature of the current study is the concurrent electrochemical acceleration technique combined with image processing in MATLAB, used to assess the evolution of concrete damage. We aim to achieve our goals by the following:

1. Crack Control Mechanism of SBC: Instead of investigating durability issues only [42], we conducted quantitative tests regarding the delay time and crack propagation rate between SBC and PC when the two are subjected to equivalent electrochemical loading.
2. Reference Data for Mesoscale Modeling: The necessary high-quality data (time series data on crack width, crack length, and crack density) needed to calibrate fracture simulations through 3D modeling techniques like the LDPM and FDEM were supplied, which do not yet have any integrated data validation with respect to electromechanics [41].
3. Empirical Database for Driven Assessment: A highly accurate empirical foundation for training future physics-aware neural networks was developed, which requires simultaneous electrochemical and mechanical boundary conditions for modeling structural deterioration [42].

## 3. Research Methodology

**Figure 1:**
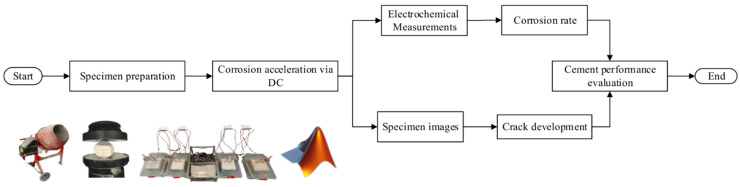
Experimental framework.

To investigate the corrosion behavior of RC containing different types of cement, a multistage experimental framework was designed to allow direct comparison between materials under controlled conditions. After selection of materials, an identical mix design was used across all samples (e.g., aggregate type, size, distribution, and water-to-cement (w/c) ratio). Cement type was designated as the only variable. This ensured that any observed differences between PC and SBC could be attributed to the binder under identical curing and exposure conditions. After casting, steel-reinforced concrete samples were cured. The next step involved accelerating corrosion through a DC process in a high-chloride environment. During the testing period, mechanical performance, electrochemical parameters, and surface deformation were monitored for structural responses and observable damage. A digital image-processing method was then developed in MATLAB to quantify this damage. At defined intervals, high-resolution images of the sample surfaces were taken and modeled to physical dimensions. Applying adaptive thresholding and area selection methods, the algorithm isolated crack patterns from the concrete background. This process served to reduce the influence of lighting variations and surface irregularities. While such an approach allows for geometric measurement of crack development, it also enables comparison of damage tolerance and cracking behavior across varying cementitious matrices. As illustrated in Figure 1, these stages were combined into a single experimental procedure, which can be used to evaluate corrosion resistance and mechanical integrity of both PC and SBC.

## 4. Experimental Program and Procedures

### 4.1. Specimen Preparation

**Figure 2:**
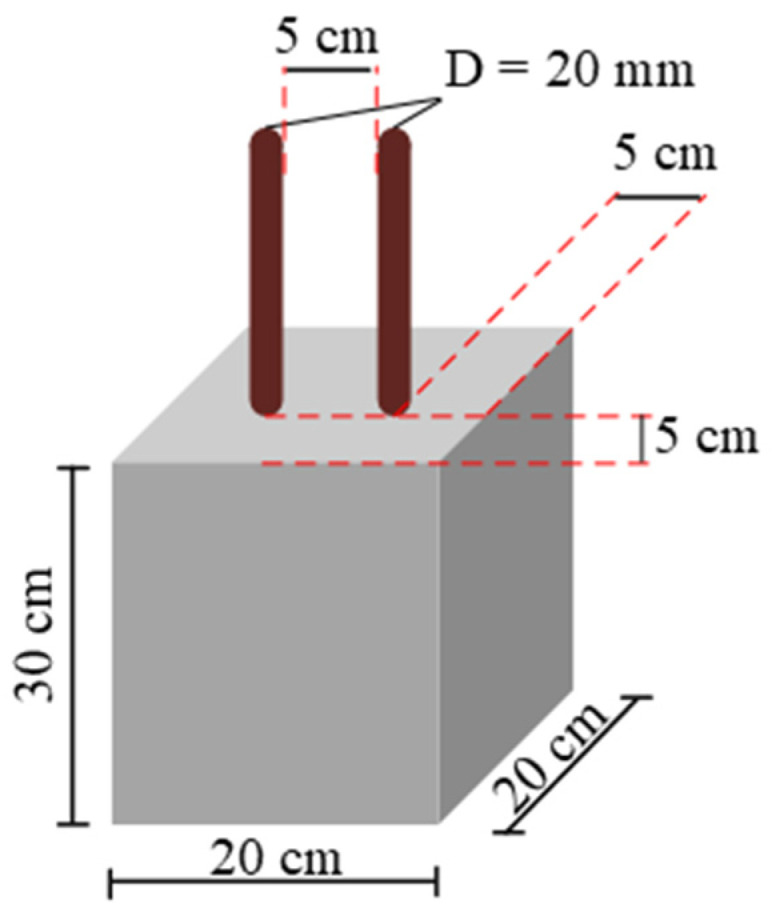
Specimen dimensions.

Two concrete mixtures were prepared for this study. One incorporated PCwhile the other was based on SBC as a more sustainable alternative (both materials were supplied by Readymix Industries Ltd, Ramat Gan, Israel). Both mixtures had identical aggregate size distributions to allow for consistency across samples. Each RC sample contained 825 kg of crushed gravel (9.5–14 mm), 775 kg of crushed sand (0.15–4.75 mm), and 322 kg of natural sand (0.15–0.6 mm), with these proportions used for both mixtures. The w/c ratio was set at 0.54 for comparable workability and strength development. The only variable between the mixtures was type of cement. This allowed for comparison of durability and corrosion resistance under identical curing and exposure conditions. To evaluate corrosion behavior under accelerated conditions, eight RC samples were cast. Four specimens were prepared from each mixture. Each sample was reinforced with two 20 mm-diameter steel bars. The specimens were all kept in the same curing room for 28 days to ensure uniform hydration and strength development. The specimens’ dimensions are presented in Figure 2.

### 4.2. Experimental Set-Up

**Figure 3:**
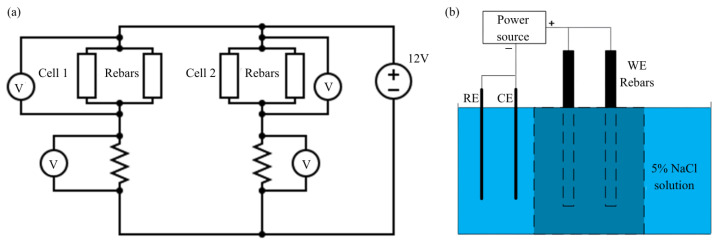
DC experimental setup (**a**) and electrochemical cell configuration (**b**).

The samples were placed in a tank filled with water having a concentration of 5% NaCl for 7 days of curing. Testing was done at 65 days after casting of concrete. The use of this method ensures that the chloride ions penetrate inside the sample material. The direct current test circuit depicted in Figure 3 consisted of two major circuits, each powered by a 12-volt DC power source, a voltmeter, and a resistor. For each circuit, the embedded steel bars acted as Working Electrode (WE). A stainless-steel rod served as the corresponding Counter Electrode (CE), resulting in a two-electrode cell. A potential difference was applied directly between the WE and CE. To determine the applied current density, the anodic surface area was defined strictly by the embedded length of the two steel bars (25 cm), excluding the exterior protruding portions. Given that the two bars were connected in parallel to make up one working electrode, the current measured was considered uniformly distributed over the surface area of the two bars. The average current density was determined by dividing the current passing through the bars by the surface area of the two bars. Under these conditions, the rebar corroded in a controlled chloride-rich environment. At the same time, voltage and current were measured consistently. This configuration was thus used to initiate and monitor time-dependent corrosion in the samples. It also enabled comparison of the durability performance of PC and SBC under identical exposure conditions.

### 4.3. Quantitative Image Analysis and Crack Characterization

To evaluate the evolution of damage, MATLAB version R2025b was used to develop a method for replacing qualitative visual inspection with quantitative measurements. As a result, measurement bias was reduced. Similar approaches involving image processing have been utilized in materials science in quantifying observations made in the laboratory, demonstrating the ability of MATLAB and other computational methods to achieve this goal [43]. Our proposed method consists of several stages. Finally, the crack widths are computed by measuring the shortest orthogonal distance between each point on the medial axis and its nearest crack edge using the Euclidean distance transform (bwdist). This produces a crack profile of varying widths, and the total crack length is determined based on the network of connected skeletons. Next, to address non-uniform illumination and surface texture variability, a supervised adaptive thresholding strategy was used via the adaptthresh function. This included an interactive interface with side-by-side comparison of the original Red, Green and Blue (RGB) image and the generated binary mask. With such an interface, sensitivity and neighborhood size parameters can be iteratively adjusted. Adjustments can thus be made until the binary image aligns with the observed crack pattern (sensitivity used ranging from 0.4 to 0.52, neighborhood size picked varied as an area of 25 to 101 pixels). At this point, structural cracks can be distinguished from surface irregularities. Following this stage, morphological filtering (using the bwareaopen function) is used to reduce noise and remove artifacts by eliminating binary objects smaller than 10 pixels. Geometric properties of the crack network are then extracted using skeletonization, thereby reducing cracks to their medial axis. Lastly, the crack width is obtained through computing the orthogonal distance of the shortest path between any point along the medial axis and the closest crack edge based on the Euclidean distance transform (bwdist). The resulting output is a width field map, whereby the crack length is obtained based on the skeleton network connections. As such, this method produces important durability indicators, e.g., maximum crack width, average crack width, and total crack length. Comparison of damage development across different concrete matrices is thus attained. To ensure reliability, the automated measurements were validated against manual measurements at selected target locations using ImageJ version 1.54r software.

## 5. Results and Discussion

The Unconfined Compressive Strength (UCS) and Brazilian Disc (BD) tests were performed on both PC and SBC mixes at days 7, 28, and 65 after mixing to assess the strength of both samples over time. Both materials showed comparable strength behavior under similar curing conditions. This information is summarized in Table 1 below. For each testing age and mixture type, the measurements were performed in duplicate. The deviation of the individual measurements from the reported mean values did not exceed 2%. While PC exhibited faster strength development at early stages, as indicated by the substantially higher 7-day strength values in Table 1, SBC showed continued strength gains at later stages. In addition, on day 65, tensile strength was evaluated using the BD test prior to the DC experiment to assess the integrity of the samples. The results indicate that both PC and SBC samples reached their expected strength levels along with similar mechanical behavior. Therefore, we predict that the different corrosion properties are caused by the increased porosity and longevity of the two types of cement used, and not by any differences in their physical properties.

### 5.1. Electrochemical Corrosion Monitoring (DC Testing)

**Figure 4:**
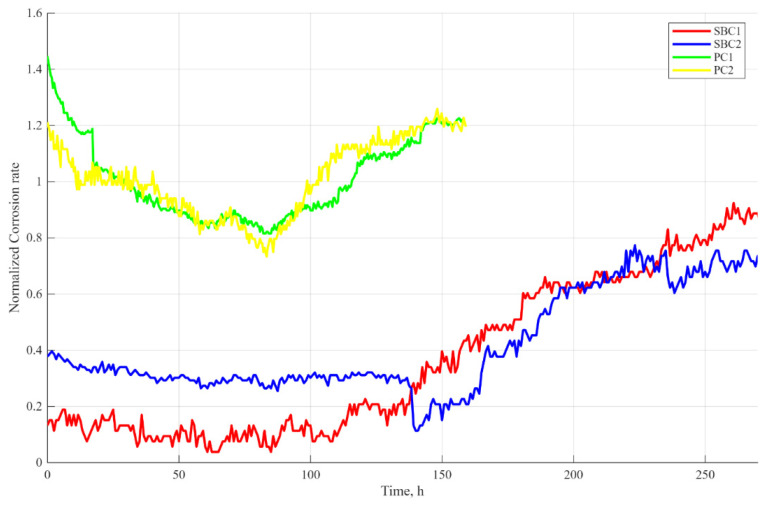
Time-dependent variation in cell normalized corrosion rates of PC and SBC specimens under accelerated DC testing.

**Figure 5:**
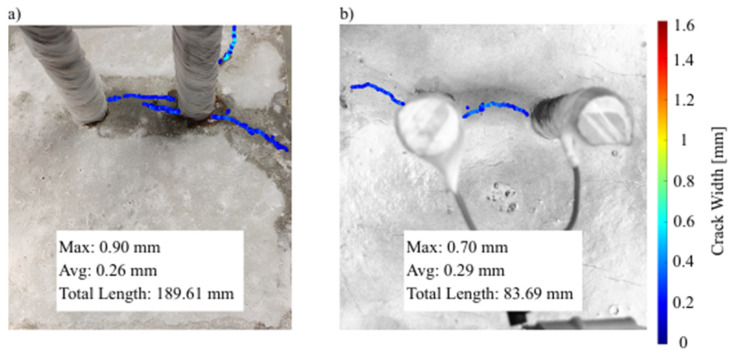
Surface cracking maps after 126 h: (**a**) PC sample and (**b**) SBC sample.

The results for two specimens out of each mixture (PC and SBC) are presented in Figure 4. Overall, the patterns of behavior, such as the delay of cracking in the case of the SBC sample, were the same in all other specimens as well. As shown in Figure 4, time-dependent normalized corrosion rates of PC and SBC samples show distinct stages of crack development and electrochemical behavior. The normalized rate is defined as the ratio of the instantaneous corrosion current (Icorr) to the average corrosion current (Icorr, average) measured over the monitoring period. In the PC samples, an initial passivation phase is observed for up to 17 h. Corrosion rates tend to slow down during this stage, indicating that corrosion products may be filling the existing pore structure adjacent to the rebar rather than increasing active structural degradation. After this stage, the corrosion rates tend to stabilize. This stability in corrosion rates may be attributed to the fact that corrosion products have blocked the already-formed pores, increased tortuosity and reduced permeability. The chronological milestones for damage evolution were identified based on distinct features in the normalized current curves. The time for crack initiation was recorded at 57 h, signifying the beginning of the depassivation process. This is followed by the phase where a high corrosion rate, along with crack propagation and rapid corrosion, is experienced at 70 h. The crack propagation stage, where high variability in the value of the current is witnessed, is seen at 83 h, as the crack network develops completely. In comparison, the SBC specimens show a late passivation and stabilization effect, producing only slight variations and a slower crack formation, with no apparent signs of deterioration. However, only after 141 h is there a noticeable change, which suggests unstable crack growth similar to that of PC, albeit at a much later period. Two representative stages from the experiment following the formation of the crack network are presented in Figure 5: The first stage shows cracking between the rebars, whereas the second stage illustrates a phase of highly developed corrosion activity.

### 5.2. Visual Comparison of Crack Propagation at Identical Corrosion Exposure

**Figure 6:**
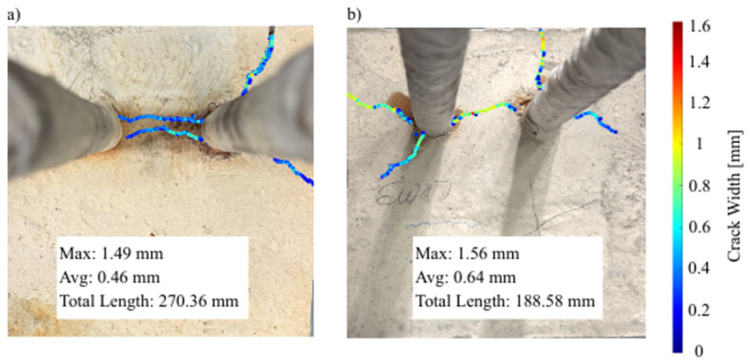
Surface cracking map at the termination of the test: (**a**) PC sample at 159 h (Q = 11.5 [Year∙µA/cm^2^]), and (**b**) SBC sample at 270 h (Q = 10.0 [Year∙µA/cm^2^]).

A direct comparison of surface crack development in the two concrete matrices under identical exposure conditions is shown in Figure 5. Both images were captured after 126 h of DC testing; by isolating the time variable, this comparison highlights the different mechanical responses of the two binders. Figure 5 and Figure 6 correspond to a typical sample with the median crack damage. Other replicate samples showed very consistent crack patterns, with variations in measured parameters not exceeding 6%. The PC sample demonstrated substantial crack damage after 126 h, when the maximum crack width was 0.90 mm, the average width was 0.26 mm, while the total crack length was 189 mm. However, the slag-based composite sample demonstrated less developed cracks at the same moment of time. The corresponding maximum crack width was 0.70 mm, the average width was 0.29 mm, and the total crack length was 83 mm. Therefore, the decrease in total crack length is about 56%, which indicates more sustainable resistance against the development of cracks.

For the reference material PC, cracks started developing around t ≈ 83 h. Thus, the post-cracking interval was set to 76 h to enable the full development of the crack network, with the final duration of the experiment equaling 159 h. Regarding the SBC material, cracks developed later due to higher resistivity, and cracks started appearing in the cover layer after t ≈ 141 h, i.e., almost twice later than for the Portland cement (141/83 ≈ 1.7). To ensure equivalence, the time scale was changed according to the same factor, meaning 76 × 1.7 = 130 h, and the duration of the experiment for the slag-based composite was equal to 270 h.

**Figure 7:**
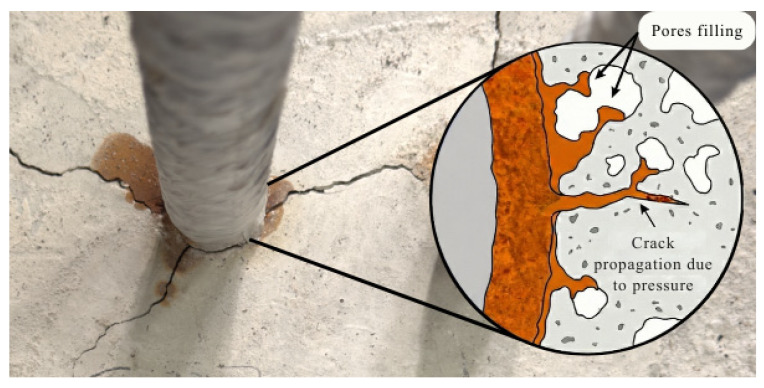
Schematic illustration of corrosion products filling pores and crack propagation due to expansive pressure.

The test conclusions for both cement types are shown in Figure 6, which represents the final damage state of the samples. The quantitative results for the slag-based binder show improved durability even with longer exposure duration and being subjected to aggressive conditions (an additional 111 h for SBC). Electrochemical monitoring supports this finding. By summing up the measured corrosion rate over the full exposure period, the total accumulated charge (Q) was obtained. It provides an indicator of total corrosion activity and was lower in SBC than in PC. In addition, there was a clear link between electrochemical behavior and resulting cracking morphology. As can be seen in Figure 6, the PC sample’s total crack length (270 mm) was much greater than the SBC (188 mm). However, this increased crack length does not translate into a more even stress distribution. Instead, it suggests increased crack coalescence and branching within localized regions. Cracks, therefore, increasingly spread and connect with one another due to the PC matrix’s relative porosity. This, in turn, results in a dense network that undermines structural continuity. In contrast, the SBC matrix exhibits a form of crack control. Even with longer exposure time and modestly larger individual crack widths (maximum: 1.56 mm), the total crack length remains lower. This suggests that cracks spread and branch to a lesser extent. As such, this is consistent with a lower rate and reduced expansive volume of corrosion activity over time. Figure 7 shows how corrosion develops and forms cracking. Because of the dense microstructure of the SBC, there is less space available for corrosion to expand into than in PC. In other words, the expansive forces produced by corrosion are more evenly distributed within the material structure. While PC causes localized damage, SBC causes cracking that is more uniformly distributed throughout the structure.

### 5.3. Validation of the Experimental Results

In order to allow comparisons with a theoretical model that assumes a constant corrosion rate, the data were normalized. During the accelerated test, the corrosion current showed variations over time. Therefore, the corrosion rate cannot be used as an accurate measure of damage development. On the contrary, corrosion damage is related to the total amount of steel consumed. Thus, the accumulated charge at crack initiation was considered the key parameter. The accumulated charge was then converted into a crack initiation time using a constant current density of 1000 µA/cm^2^. This resulted in a standardized basis for comparison with the theoretical model. This normalized experimental time was then compared to the analytical prediction model proposed by Lu et al. This model assumes that the concrete around the reinforcement acts as a thick-walled cylinder and applies the theory of elasticity to correlate the radial pressure caused by expansion with the weight loss in the corroded steel. This model also accounts for the presence of corrosion products within the porous region adjacent to the steel surface and within radial cracks [8]. The theoretical time to cover cracking was calculated using the following equation [8]:

(1) $$\begin{array}{l} t_{cr}\\ =234762\left(d+kc\right)\\ \cdot \frac{{\left(\left(0.3+0.6\frac{c}{d}\right)\cdot \frac{f_{ct}}{E_{cef}}\cdot \left[\frac{{\left(r_{0}+c\right)}^{2}+r_{0}^{2}}{{\left(r_{0}+c\right)}^{2}-r_{0}^{2}}+\nu_{c}\right]+1+\frac{2\delta_{0}}{d}\right)}^{2}-1}{(n-1)\cdot I_{corr}} \end{array}$$

where *d* represents the original diameter of the reinforcing bar (20 mm) and *k* is the modified coefficient that depends on the corrosion condition of the steel and is set to 0.2 for accelerated corrosion. The geometric and material parameters include the concrete cover thickness, *c* (50 mm), tensile strength, *f_ct_* (see Table 1), and effective elastic modulus, *E_cef_*, which is derived from the compressive strength using the equation $E_{cef}=4500\sqrt{f_{c}^{\prime }}$ [8]. Additionally, *δ*_0_ is the thickness of the porous zone surrounding the rebar, *r*_0_ denotes the effective inner radius calculated as *r*_0_ = *d*/2+ *δ*_0_, and *ν_c_* is Poisson’s ratio (assumed to be 0.2). Finally, *n* represents the ratio of the volume of corrosion products to the volume of consumed iron (assumed 2.7). The model was adjusted to reflect the microstructural characteristics of each binder. For the PC reference, a porous zone thickness of *δ*_0_ = 0.017 mm was used. This value represents a relatively porous interfacial region. In contrast, for the SBC matrix, a lower bound value of *δ*_0_ = 0.012 mm was adopted. This is based on microstructural studies [14] of slag-blended cements. These confirmed that the ITZ in SBC is denser, and so there are fewer pores where corrosion products can accumulate. With these parameters, the predicted cracking time for the PC samples was 15.05 h, which closely matches the normalized experimental value of 14.9 h. In the SBC matrix case, the estimated crack initiation time is 11.34 h, showing an error of around 1% compared to the measured value of 11.4 h. In general, theoretical analysis is in good agreement with the normalized experimental results. Since the SBC matrix has a smaller porous layer (*δ*_0_), the analysis shows that less energy is required to initiate the crack. However, this indicates that the delayed cracking of the SBC matrix is mainly influenced by its low electrical conductivity, leading to lower resistivity. Finally, n represents the ratio of the volume of corrosion products to the volume of consumed iron (assumed 2.7). The model was adjusted to reflect the microstructural characteristics of each binder. For the PC reference, a porous zone thickness of *δ*_0_ = 0.017 mm was used. This value represents a relatively porous interfacial region. In contrast, for the SBC matrix, a lower bound value of *δ*_0_ = 0.012 mm was adopted. This is based on microstructural studies [14] of slag-blended cements. These confirmed that the ITZ in SBC is denser, and so there are fewer pores where corrosion products can accumulate. With these parameters, the predicted cracking time for the PC samples was 15.05 h, which closely matches the normalized experimental value of 14.9 h. In the SBC matrix case, the estimated crack initiation time is 11.34 h, showing an error of around 1% compared to the measured value of 11.4 h. In general, theoretical analysis is in good agreement with the normalized experimental results. Since the SBC matrix has a smaller porous layer (*δ*_0_), the analysis shows that less energy is required to initiate the crack. However, this indicates that the delayed cracking of the SBC matrix is mainly influenced by its low electrical conductivity, leading to lower resistivity.

## 6. Conclusions

After 65 days, a comparative analysis of PC and SBC showed equivalent compressive and tensile strengths in both materials. Thus, the difference in behavior towards corrosion is mainly associated with the material’s durability rather than its initial bearing capacity. The following conclusions were reached from the experimental work. The electrochemical study, along with the automated crack width measurement, showed excellent corrosion resistance properties in SBC. Initial deterioration in the PC samples was observed at the widest crack, which measured 1.49 mm. On the other hand, crack initiation in SBC showed a significant delay from 70 to 141 h.

Moreover, quantitative image analysis confirmed the advantage of the SBC binder in this respect: a 56% decrease in crack length was observed in both materials after 126 h. By the end of the experiment, a clear difference in crack-control ability was confirmed: the total crack length was 30% lower than in PC specimens. The results presented show that the slag matrix has a better capacity to distribute stress. The suggested evaluation method highlights the enhanced crack-control mechanism of SBC when used in aggressive environments, as validated. In summary, the developed approach successfully bridges the gap between electrochemical corrosion and mechanical damage, providing a highly correlated empirical dataset that serves as a promising basis for evaluating alternative binders through accelerated electrochemical analysis and crack assessment.

## 7. Future Work

This study provides a basis for future work in which mesoscale fracture models (LDPM/FDEM) can be calibrated against these experimental results, thereby creating a digital representation of the corrosion process. Despite the fact that the current experiment involved eight samples spread across two mixture designs, generalized calibration requires variation to be systematic. Therefore, the next steps for further investigation should focus on increasing the database by adding more variables, such as binder composition, cover thickness, reinforcing bar diameter, reinforcing bar configuration, and environmental variables. Moreover, microstructural and chemical analyses, such as measuring the pH of the pore solution and evaluating electrical resistivity, will provide greater insight into corrosion processes. Once validated, these simulations can be used to create a synthetic dataset to train physics-based AI models to predict structural behavior due to corrosion. Overall, this study lays a foundation for future assessment of advanced cementitious systems and the development of large datasets needed to move from empirical observation toward AI-driven predictive maintenance, demonstrating how experimentally grounded datasets are essential for calibrating the numerical models that feed deep-learning-based damage localization.

## Figures and Tables

**Table 1:** Mechanical strength measured.

Test Type	Day	PC	SBC
Compressive	7	43.7	33.3
Compressive	28	55.3	51.9
Compressive	65	60.7	60.0
Tensile	65	4.3	4.5

## Data Availability

The original contributions presented in this study are included in the article. Further inquiries can be directed to the corresponding author.

## Abbreviations

The following abbreviations are used in this manuscript:

PC	Portland Cement
SBC	Slag-Blended Cement
DC	Direct Current
RC	Reinforced Concrete
LDPM	Lattice Discrete Particle Method
FDEM	Finite Discrete Element Method
DIC	Digital Image Correlation
GGBS	Ground Granulated Blast-furnace Slag
C–S–H	Calcium Silicate Hydrate
C3A	Tri-Calcium Aluminate
AFm	Aluminate Ferrite mono-substituent
ITZ	Interfacial Transition Zone
CH	Calcium Hydroxide
MATLAB	Matrix Laboratory
NDE	Nondestructive Evaluation
AE	Acoustic Emission
IE	Impact-Echo
PINN	Physics-Informed Neural Network
AI	Artificial Intelligence
WE	Working Electrode
CE	Counter Electrode
RGB	Red, Green and Blue
UCS	Unconfined Compressive Strength
BD	Brazilian Disc
MPa	MegaPascal
mm	millimeter
µA/cm^2^	MicroAmperes per square centimeter
